# Get the most out of blow hormones: validation of sampling materials, field storage and extraction techniques for whale respiratory vapour samples

**DOI:** 10.1093/conphys/cow024

**Published:** 2016-08-26

**Authors:** Elizabeth A. Burgess, Kathleen E. Hunt, Scott D. Kraus, Rosalind M. Rolland

**Affiliations:** John H. Prescott Marine Laboratory, New England Aquarium, 1 Central Wharf, Boston, MA 02110, USA

**Keywords:** Blow, cetacean, hormone analysis, technique validation

## Abstract

Studies are progressively showing that vital physiological data may be contained in the respiratory vapour (blow) of cetaceans. Nonetheless, fundamental methodological issues need to be addressed before hormone analysis of blow can become a reliable technique. In this study, we performed controlled experiments in a laboratory setting, using known doses of pure parent hormones, to validate several technical factors that may play a crucial role in hormone analyses. We evaluated the following factors: (i) practical field storage of samples on small boats during daylong trips; (ii) efficiency of hormone extraction methods; and (iii) assay interference of different sampler types (i.e. veil nylon, nitex nylon mesh and polystyrene dish). Sampling materials were dosed with mock blow samples of known mixed hormone concentrations (progesterone, 17β-estradiol, testosterone, cortisol, aldosterone and triiodothyronine), designed to mimic endocrine profiles characteristic of pregnant females, adult males, an adrenal glucocorticoid response or a zero-hormone control (distilled H_2_O). Results showed that storage of samples in a cooler on ice preserved hormone integrity for at least 6 h (*P* = 0.18). All sampling materials and extraction methods yielded the correct relative patterns for all six hormones. However, veil and nitex mesh produced detectable assay interference (mean 0.22 ± 0.04 and 0.18 ± 0.03 ng/ml, respectively), possibly caused by some nylon-based component affecting antibody binding. Polystyrene dishes were the most efficacious sampler for accuracy and precision (*P* < 0.001), but required an ethanol rinse for improved progesterone recovery (increased 81%; *P* < 0.001). Awareness of assay interference from exogenous materials is crucial to future studies. This study establishes critical groundwork to help ensure that hormones can be measured accurately in samples obtained from field collections of whale blow.

## Abstract (precis)

Respiratory vapor (blow) of cetaceans may contain vital physiologic data, yet fundamental methodological issues remain to be addressed for this nascent technique. We validated sample field storage; hormone extraction methods; and assay interference from sampling materials in order to ensure that reliable hormone data is obtained from whale blow.

## Introduction

As marine ecosystems are increasingly impacted by human activities, there is an urgent need to develop novel techniques for physiological assessment of living whales (Hunt *et al*., 2013). Endocrine information, in particular, can afford managers some insight into biological processes of conservation concern, namely reproduction and stress responses (Pukazhenthi and Wildt, 2004; Wikelski and Cooke, 2006). A major challenge in assessing large whales in this manner is that sample types conventionally used for hormone analysis (i.e. blood) are logistically impossible to collect from free-swimming individuals. However, whales breathe at the surface with huge tidal volumes (see Piscitelli *et al*., 2013), and the exhaled respiratory vapour or ‘blow’ may have a chemical composition that reflects components circulating in blood (see Aksenov *et al*., 2014), presenting a promising approach for non-invasive sampling of internal physiology.

Field studies have shown that respiratory samples can be collected from whales by positioning a sampling device above (~0.5–1 m) the exhaling blowholes to catch a portion of the aerosol droplets (Hogg *et al*., 2009; Hunt *et al*., 2014a). Various collection materials have been used for this purpose, including nylon stocking, nylon veil, nylon mesh, cotton gauze, polypropylene containers and Petri dishes (Hogg *et al*., 2005, 2009; Acevedo-Whitehouse *et al*., 2010; Hunt *et al*., 2014a; Thompson *et al*., 2014). Moreover, several classes of steroid and thyroid hormones have been successfully detected in blow samples from North Atlantic right whales (*Eubalaena glacialis*; Hogg *et al*., 2009; Hunt *et al*., 2014a) and humpback whales (*Megaptera novaeangliae*; Hogg *et al*., 2009), as well as smaller cetaceans in captivity or under restraint in the wild, namely bottlenose dolphins (*Tursiops truncatus*; Hogg *et al*., 2005; Aksenov *et al*., 2014) and belugas (*Delphinapterus leucas*; Thompson *et al*., 2014). Recent analytical developments have shown that convenient and commercially available enzyme immunoassays are sensitive enough (picogram per millilitre level) to detect blow hormones (Hunt *et al*., 2014a; Thompson *et al*., 2014). Although sampling whale blow at sea can be challenging, it has proved feasible to collect samples from targeted individuals, as well as to obtain repeated samples over time (Hogg *et al*., 2009; Hunt *et al*., 2014a). These major sampling benefits, along with the prospect that hormone samples may provide insight into a range of biological questions (see Hunt *et al*., 2013), have encouraged researchers to begin exploring blow hormones in a number of cetacean species (e.g. Dunstan *et al*., 2012). However, important methodological issues still need to be addressed before quantification of hormone concentrations in blow samples can become a reliable technique (see Trout, 2008; Hunt *et al*., 2014a).

The validity of hormonal analysis depends on accurate measurement of the hormone concentration in the designated sample matrix. Generally, most hormones are present in all individuals (of both sexes) at all times; what matters is the quantified concentration and/or relative levels in association with biological factors of interest. Biological variations in the concentrations of reproductive hormones may reflect sexual maturity, reproductive seasonality and/or the reproductive state of individuals (e.g. testosterone, progesterone and estradiol), whereas thyroid and adrenal hormones may reflect nutritional and metabolic processes (e.g. triiodothyronine) and/or activation of the stress system (e.g. cortisol and aldosterone; Norris and Carr, 2013). However, technical factors can play a crucial role in the successful evaluation of hormone concentrations. Fundamental concerns that may affect the resulting sample concentrations include the following: whether field storage conditions adequately preserve the sample hormone after collection (see Woods, 1975; Millspaugh and Washburn, 2004; Ziegler and Wittwer, 2005); the efficiency of the technique used to extract hormones from the sample (see Lynch *et al*., 2003); and whether there is assay interference from exogenous materials used in the collection and/or extraction process (e.g. cotton used in blow sample collection has been shown to interfere with some immunoassays; Hogg *et al*., 2009; Thompson *et al*., 2014). For blow hormone analysis, these technical considerations are especially relevant because of the relatively low concentrations of exhaled hormone metabolites and the need for a large surface area to collect respiratory droplets, as well as unknown variation in the whale's respiratory vapour flow.

Currently, there is no consensus about the best procedures or precautions for blow sample storage in the field or sample preparation prior to hormone analysis. Evaluation of the factors that may distort or bias hormone concentrations is necessary to demonstrate the validity of any new method and must be achieved before a new technique merits general acceptance by the research community. In this study, we investigated the following technical issues pertaining to sample handling and processing (i.e. after collection and before immunoassay) that might influence the integrity of cetacean blow hormone results: (i) typical field storage of samples during daylong trips on small boats (i.e. insulated cooler with samples on ice); (ii) efficiency of hormone extraction methods; and (iii) assay interference from sampling and processing materials. Overall, our objective was to lay the foundation for application of hormone analysis of respiratory vapour to cetacean field research.

## Materials and methods

### Sampler types

The following three materials were experimentally tested as potential sampler types for collecting whale respiratory vapour: (i) commercial nylon veil (ordinary bridal tulle fabric; ‘veil’ hereafter); (ii) laboratory-grade nitex nylon 110 mm mesh (Elko Filtering, Miami, FL, USA; ‘nitex mesh’ hereafter); and (iii) sterile polystyrene dishes (Corning^®^ square 25 cm × 25 cm, CLS431111; Sigma-Aldrich, St Louis, MO, USA; ‘dish’ hereafter). To date, nylon (veil or stocking fabric) has been the most field-tested collection material for large whale blow (Hogg *et al*., 2009; Hunt *et al*., 2014a). However, more recent collection trials, using blow from captive cetaceans, demonstrated that nitex mesh had an improved performance in retaining sample volume (cf. nylon veil and cotton gauze), with reduced assay interference (cf. nylon stocking; Thompson *et al*., 2014). An alternative methodological approach is to avoid using fabric by collecting blow droplets onto a non-permeable surface, such as a polystyrene dish (see Acevedo-Whitehouse *et al*., 2010). Each of these materials requires modifications of sample processing and hormone extraction, which could potentially influence the resulting hormone concentrations. Our study design uses a holistic comparison of each sampler type (i.e. encompassing the sampling material, its associated outer storage container and an extraction protocol; see ‘*Extraction techniques*’ below) in a controlled experimental setting.

For preparation of nylon sampler types, veil material was cut to 90 cm × 180 cm (folded 45 cm × 45 cm in eight-ply), and nitex mesh material was cut to 30 cm × 30 cm (single ply). Both veil and nitex mesh materials were thoroughly washed before use, using multiple wash cycles (Hogg *et al*., 2009; Hunt *et al*., 2014a) that involved soaking in warm soapy water for 10 min, then rinsing with tap water to remove soap, rinsing with distilled water (dH_2_O) twice, submerging and agitating in 70% ethanol (EtOH) for 15 min, and air drying. Nylon samplers were sealed in individual clean zip-type plastic bags, ready for use. Dishes had a clean, sterilized surface and lid that did not require any washing before experimental treatment.

### Experimental design and hormone treatments

To determine the efficiency of hormone recovery from each sampler type, we prepared three different solutions with varying hormone ratios designed as mock ‘blow’ solutions containing a mixture of several different hormones, in addition to a fourth control solution consisting of dH_2_O only (i.e. no added hormone). Mixed hormone solutions were designed to mimic the general pattern of three hormone profiles of interest (adult male, pregnant female and adrenal glucocorticoid response), as well as to simulate a variety of hormone ratios across solutions. Hormone concentrations were prepared within a range of 0.1 (low) to 10 ng/ml (high) (Table 1), which is representative of serum concentrations in large whales (e.g. fin whale, *Balaenoptera physalus*, progesterone range 0.2–12 ng/ml; testosterone range 0.03–12 ng/ml; estradiol mean range 0.02–12 ng/ml; Kjeld *et al*., 2006). Preliminary data from cetacean blow in both mysticetes and odontocetes indicates that blow is likely to have similar hormone concentration ranges to plasma (Hunt *et al*., 2014a; Thompson *et al*., 2014).

**Table 1:** Actual hormone concentrations (in nanograms per millilitre ± SD) in experimental treatment solutions (control, adult male, pregnant female and adrenal glucocorticoid response profiles) prepared as low (~0.1 ng/ml; light shade), medium (~1 ng/ml; medium shade) and high concentrations (~10 ng/ml; dark shade) of various hormones [testosterone (T), progesterone (P4), estradiol (E2), cortisol (F), aldosterone (ALD) and triiodothyronine (T3)]

	Hormone concentration (ng/ml)					
Treatment solution	T	P4	E2	F	ALD	T3
Control	0.0 ± 0.0	0.0 ± 0.0	0.0 ± 0.0	0.0 ± 0.0	0.0 ± 0.0	0.0 ± 0.0
Adult male	11.5 ± 0.9	0.0 ± 0.0	0.1 ± 0.0	8.2 ± 0.7	0.8 ± 0.1	0.8 ± 0.1
Pregnant female	0.2 ± 0.0	8.5 ± 0.2	0.7 ± 0.1	0.6 ± 0.1	0.9 ± 0.2	0.6 ± 0.1
Adrenal glucocorticoid response	0.3 ± 0.0	0.0 ± 0.0	0.7 ± 0.0	8.4 ± 0.6	11.1 ± 1.5	0.7 ± 0.1
Assay limit of detection	0.03	0.05	0.03	0.05	0.01	0.07

For preparation of these treatment solutions, pure crystalline progesterone (catalogue no. P0130), 17β-estradiol (catalogue no. E8875), testosterone (catalogue no. T1500), cortisol (catalogue no. H4001), aldosterone (catalogue no. A9477) and triiodothyronine (catalogue no. T2877) were used (all from Sigma-Aldrich, St Louis, MO, USA). Stock solutions of each hormone were prepared in HPLC-grade EtOH and stored in Pyrex^®^ 100 ml glass bottles. Final treatment solutions were then prepared in dH_2_O using the stock solutions to produce the desired combination of concentrations that were near the intended high (10.0 ng/ml), medium (1.0 ng/ml) or low (0.1 ng/ml) concentrations of each hormone (see Table 1). Triiodothyronine was the only hormone added at a uniform concentration to all mixed hormone solutions, because this hormone often has a narrow range of concentrations among individual cetaceans (e.g. St Aubin *et al*., 1996), especially when compared with steroids. Final concentrations of treatment solutions were expected to have minor deviation from target concentrations (see Table 1), as is typical when preparing hormone standards at the nanogram to picogram per millilitre level.

In the experiment, the surface of each sampling material was dripped with 1.0 ml of a treatment solution (i.e. mixed-hormone ‘blow’ solution or control dH_2_O), simulating capture of respiratory vapour droplets from a whale. Previous field trials revealed that 1.0 ml is a typical ‘high-quality’ sample volume collected from a single exhalation of a large whale and that respiratory vapour samples from large whales typically are highly aqueous, with no visible lipid or mucoid fraction, even after centrifugation (Hunt *et al*., 2014a). For each treatment, we conducted eight replicates for each sampler type [*n* = 4 treatments (three mixed hormone solutions plus one control) × 8 replications = 32 samples for each sampler type]. This experiment was done in duplicate for the dish samplers in order to test two different methods of extracting (recovering) hormone from a flat dish surface (i.e. pipetting off droplets vs. rinsing the dish with 100% EtOH; (see ‘*Extraction techniques*’ below). Once treated with the mock ‘blow’ solution or control dH_2_O, the veil and nitex mesh samplers were sealed in an individual zip-type plastic bag, whereas dish samplers were covered with the lid and then sealed in a zip-type plastic bag. All samplers were then placed in a thick-walled (3.8 cm) cooler on icepacks for 6 h to simulate typical field storage conditions on a small research vessel during daylong sampling at sea. After 6 h, hormones were extracted from each sampler (total *n* = 128).

### Extraction techniques

Hormones were extracted using different methods depending on the sampling material. Veil samplers (*n* = 32) were extracted by pouring 100 ml of 100% EtOH over each veil inside a 473 ml glass jar, the minimal size of container that allowed complete submergence of the eight-ply fabric in EtOH. The jar was sealed and hand shaken vigorously for 60 s, after which the liquid was decanted into 25 mm × 125 mm borosilicate glass tubes. These steps were repeated for a second rinse. An additional 20 ml of 100% EtOH was used to rinse the inside of the zip-type plastic bag that contained the sampler. The combined ~220 ml EtOH rinse (containing hormones) in glass tubes was evaporated to dryness under compressed air for 48 h and reconstituted in 1.0 ml of dH_2_O for analysis.

Nitex mesh samplers (*n* = 32) were extracted by pouring 80 ml of 100% EtOH over each nitex mesh inside a 120 ml polypropylene jar. The solvent volume for nitex mesh was lower than that for veils owing to the smaller size of the nitex mesh; in both cases, enough EtOH was added to immerse the sampling material thoroughly. The jar was sealed and vigorously mixed on a plate-shaker for 1 h, after which the liquid was decanted into 25 mm × 125 mm borosilicate glass tubes. The nitex mesh was then put into a 50 ml Falcon^®^ tube on top of two capped 2 ml microcentrifuge tubes (i.e. elevating the material off the bottom) and centrifuged at 4000 *g* for 15 min. It is noteworthy that the smaller fabric volume of the nitex mesh permitted controlled centrifugation (cf. hand-shaken veil samples, which were too large to centrifuge). Recovered fluid was added to the glass tubes. The zip-type plastic bag was also rinsed with 20 ml of 100% EtOH. The combined ~100 ml EtOH rinse in glass tubes was dried under compressed air for 24 h and reconstituted in 1.0 ml of dH_2_O.

Dish samplers do not involve a fabric (unlike nitex mesh or veils); hence, the following two extraction methods were tested: (i) direct extraction by pipetting (*n* = 32; ‘pipetted dish’ hereafter); and (ii) an EtOH rinse (*n* = 32; ‘rinsed dish’ hereafter). The pipetting method was tested because it had the potential to minimize additional sample processing steps (e.g. dry-down and reconstitution) that might introduce noise to the hormone data. Pipetting was not attempted with the veil or nitex mesh because previous field trials showed that cetacean blow hormone adheres to fabric-type samplers; Hunt *et al*., 2014a). The pipetting method involved using a 1000 µl pipette to draw up all visible droplets from the surface of the dish and transfer them into a microcentrifuge tube for later assay. For the rinse method, 50 ml of 100% EtOH was poured over the dish, which was lidded and gently agitated on a plate-shaker for 30 min (i.e. to suspend any hormone that might have adhered to the dish surface). The EtOH rinse was then decanted into 25 mm × 125 mm borosilicate glass tubes, dried under compressed air for 24 h and reconstituted in 1.0 ml of dH_2_O. All samples (total *n* = 128) were stored frozen at −20°C until hormone analysis.

Given that the containers used for storage and extraction of veil and nitex samplers were varied (mostly owing to the fabric size; see above), a supplemental experiment was conducted to investigate the influence of each container type [i.e. polypropylene (zip-type) bag, polypropylene jar and glass jar] as a source of assay interference. In brief, clean polypropylene bags, polypropylene jars and glass jars were individually rinsed with 100% EtOH (according to the extraction protocol above), and the resultant samples (*n* = 8 replicates for each container type) were analysed for progesterone, testosterone and cortisol (bracketing the range of polarity of steroids tested in this study; see ‘*Hormone assays*’ below). When tested in isolation, none of the bag or jar types used in this study produced interference effects (i.e. non-detectable hormone concentrations for all assays tested; data not shown).

### Hormone assays

Enzyme immunoassays were used to quantify concentrations of testosterone (#K032-H1), progesterone (#K025-H1), estradiol (#K036-H1), cortisol (#K003-H1) and aldosterone (#052-H1; 2 h protocol; all from Arbor Assays, Ann Arbor, MI, USA). Radioimmunoassay was used to quantify triiodothyronine concentrations (#06B-254215; MP Biomedicals, Solon, OH, USA). These commercially available assay kits were selected based on previous successful use with respiratory vapour collected from free-swimming whales (see Hunt *et al*., 2014a). Assay procedures were performed according to the manufacturer's protocols. For further assay details and antibody cross-reactivities, see the enzyme immunoassay protocols from Arbor Assays (http://www.arborassays.com) and the radioimmunoassay protocol from MP Biomedicals (http://www.mpbio.com). Samples containing 10 ng/ml concentrations of particular hormones were assayed at a 1:20 dilution in dH_2_O for that hormone's assay. Pure mixed hormone ‘blow’ solutions and pure dH_2_O were distributed equally within and across assays to minimize potential effects of inter-assay variation. To monitor precision and reproducibility in our assays, low- (70–80% binding) and high-quality (20–30% binding) control samples were run on each plate (total *n* = 5 assays performed for each hormone). All assays were performed by the same person, with all samples, controls and standards assayed in duplicate, and results averaged accordingly. Intra-assay coefficient of variation averaged 4.0% across assays, calculated from the variation of measurements between duplicates. Inter-assay coefficient of variation was <7.0% (low control) and <5.0% (high control) for all six assays. Any sample with a coefficient of variation >10% was re-assayed. Final data were expressed as nanograms per millilitre of extracted sample.

### Statistical analyses

Our analytical method focused on a comparison between apparent (measured) concentrations of samples and the known concentration of the treatment solution that had been dripped onto the sampler. The best sampling material and extraction technique would be that which demonstrated the accuracy and precision in measured hormone concentrations compared with the ‘actual’ concentration in pure solution after sample processing.

We used simple descriptive statistics (mean ± SD) to summarize the data set. All data were analysed using SPSS statistical software (version 22.0 for Macintosh; IBM Corp., Armonk, NY, USA). Hormone data were log_10_-transformed to adjust for non-normal distributions based on skewness and kurtosis. Non-detectable values were substituted with half the limit of detection for that particular assay (see Table 1), in order to allow transformation of all data. Control treatment results were used to evaluate whether any sampling material and extraction technique interfered with the assay, i.e. causing spurious apparent hormone concentrations. Two-way analysis of variance was used to assess differences in hormone concentrations between treatment solutions and between sampler types. Where significant differences were detected, *post hoc* comparisons using Tukey's HSD test were performed to identify the source of variance. Assay results of the three mixed hormone solutions were considered as the ‘actual’ (known) concentrations applied in treatments. Student's paired *t*-test was used to detect differences in hormone concentrations between the pure mixed hormone solution and the resulting sample after experimental treatment. Accuracy was evaluated as the difference between the measured hormone concentration in the resulting sample and the known concentration of the mixed hormone solution. Precision was measured by the standard deviation among samples for each sampler type. The percentage recovery of each hormone in samples was calculated from the actual concentration expected, i.e. percentage recovery = measured concentration/actual concentration × 100. Hormone recoveries from each sampler type were compared using data from high-concentration samples (10 ng/ml), because these concentrations yielded the greatest assay reliability (i.e. near 50% bound on standard curve). Results for the two different extraction techniques tested on dish samplers (EtOH rinse vs. direct pipetting) were compared using a two-tailed Student's unpaired *t*-test.

In order to evaluate the overall efficiency of each sampling material and respective extraction methods, we reduced the data for all six hormones using a multivariate principal components analysis. Before performing principal components analysis, the suitability of data for factor analysis was assessed. Inspection of the correlation matrix revealed the presence of coefficients of 0.3 and above. The Kaiser–Meyer–Olkin value was 0.5, and Bartlett's test of sphericity reached statistical significance (*P* < 0.001), supporting the factorability of the correlation matrix (Quinn and Keough, 2002). Principal components analysis revealed the presence of two components with eigenvalues exceeding 1, and an inspection of the scree plot showed a clear break after the second component. To aid in the interpretation of these two components, oblimin rotation was performed, with the two factors showing low inter-correlation (*r =* 0.21). The resulting eigenvector loadings associated with the new components were examined graphically to assess how each sampler type was able to distinguish between treatment solutions. For all analyses, *P* < 0.05 was considered as significant.

## Results

All three treatment solutions had significantly different combinations of hormones (*F*_2,72_ = 32.9, *P* < 0.001; Table 1), demonstrating that the three hormone profiles, representing a (hypothetical) adult male, pregnant female and adrenal glucocorticoid response, were statistically distinguishable from each other. The immunoassays used had adequate sensitivity to measure quantities within the range of the lowest prepared hormone concentration used in this study (0.1 ng/ml). The only exception was the progesterone assay, which produced non-detectable results (i.e. between zero and the detection limit of the assay) for the low-level progesterone concentration (0.1 ng/ml) in the adult male and adrenal glucocorticoid response solutions when assayed without dilution. Nonetheless, all immunoassay results accurately differentiated between relative patterns of high, medium and low concentrations between treatment solutions for all hormones (all *P <* 0.001); excluding triiodothyronine hormone, which was prepared as a uniform concentration across treatment groups (*F*_2,13_ = 2.79, *P* = 0.21; Table 1). Distilled water, assayed as a pure solution, had non-detectable concentrations for all hormones under study, as expected (Table 1).

**Figure 1:**
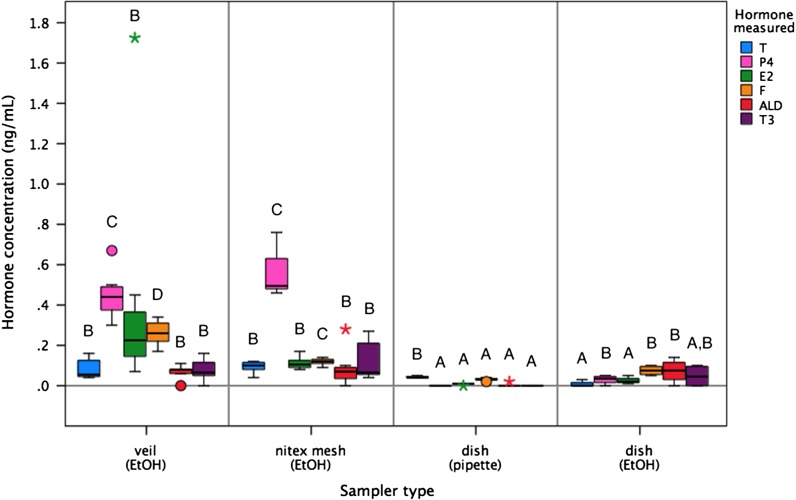
Quantification of assay interference from different sampling materials (veil, nitex mesh or dish) after extraction [using ethanol (EtOH) rinse or direct pipetting; *n* = 8 replicates for each sampler type], as determined for six immunoassays [testosterone (T), progesterone (P4), estradiol (E2), cortisol (F), aldosterone (ALD) and triiodothyronine (T3)]. For boxplots, the line inside the box indicates the median value, the height of the box encompasses the distance between the 25th and 75th quartiles, and the whiskers delineate extreme observations. Outliers are marked with a circle (>1.5 × interquartile range) and extreme outliers are marked with a star (>3 × interquartile range). Different letters denote a significant difference in resulting hormone measures between sampler types at *P* < 0.05.

All sampler types in the control treatment (no added hormone) produced some level of apparent (spurious) hormone in all immunoassays tested (Fig. 1). This measurable immunoreactivity was intrinsic to each sampler type, probably indicating interference with antibody binding in the assay. For all sampler types and hormones tested, assay interference levels averaged <0.3 ng/ml, with one extreme outlying result from a single veil sampler for estradiol (1.73 ng/ml). Levels were greatest for the progesterone assay (0.29 ± 0.26 ng/ml), followed by estradiol (0.15 ± 0.32 ng/ml) and cortisol (0.13 ± 0.10 ng/ml), and were very low for triiodothyronine (0.06 ± 0.07 ng/ml), testosterone (0.06 ± 0.04 ng/ml) and aldosterone (0.05 ± 0.06 ng/ml; *F*_5,167_ = 9.30, *P* < 0.001). However, these measures of assay interference were significantly different between sampler types (*F*_3,167_ = 24.13, *P* < 0.001; Fig. 1). The highest level of interference was measured from veil and nitex mesh samplers (mean across all hormones, 0.22 ± 0.04 and 0.18 ± 0.03 ng/ml, respectively), whereas rinsed dishes had lower levels (0.04 ± 0.01 ng/ml), and pipetted dishes demonstrated the least interference in hormone assays (0.01 ± 0.00 ng/ml). Assay interference from all sampler types and across all immunoassays demonstrated repeatability, as determined by low standard deviations between replicates (nitex mesh, SD < 0.11 ng/ml; veil, SD < 0.54 ng/ml; rinsed dish, SD < 0.06 ng/ml; and pipetted dish, SD < 0.01 ng/ml), i.e. considered to be background noise (Fig. 1).

**Figure 2:**
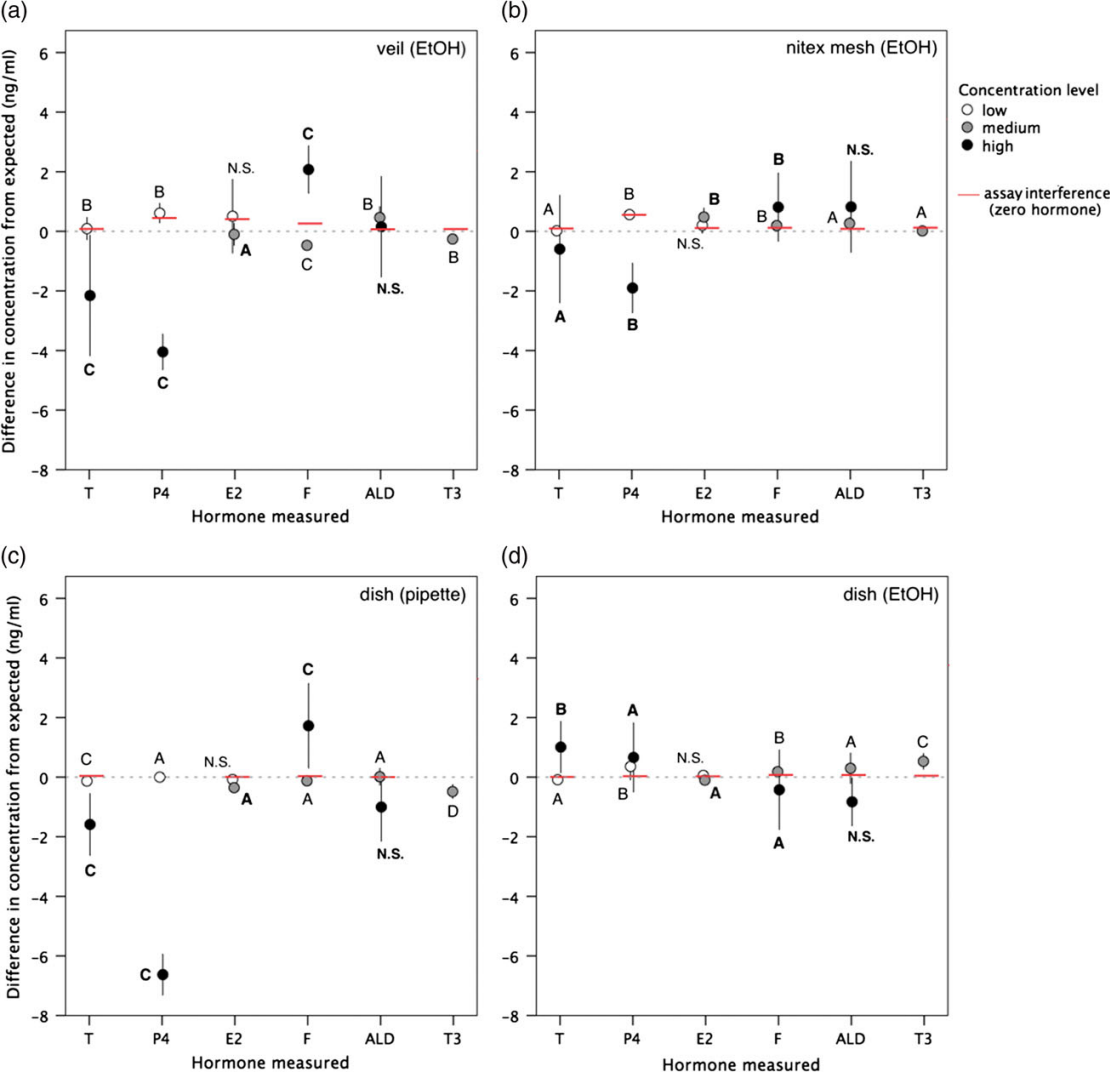
Accuracy of hormone results (observed minus expected concentration, in nanograms per millilitre) using different sampling materials (veil, nitex mesh or dish) and respective extraction methods [ethanol (EtOH) rinse or direct pipetting]. Hormones measured were testosterone (T), progesterone (P4), estradiol (E2), cortisol (F), aldosterone (ALD) and triiodothyronine (T3). Values are means (±SD) across low (~0.1 ng/ml; open circles), medium (~1 ng/ml; shaded circles) and high (~10 ng/ml; filled circles) concentrations of mixed hormone treatment solutions. Zero values indicate that the observed concentration equals the actual concentration in pure treatment solutions; negative values indicate a deficit in observed hormone concentration; and positive values indicate a surplus. Red bars indicate the detectable level of immunoassay interference (see Fig. 1). Different letters denote a significant differences in resulting hormone measures between sampler types at *P* < 0.05 (N.S. denotes no statistical significance).

After storing samplers for 6 h in a cooler, all samples yielded hormone measurements that were statistically similar to actual concentrations of the pure mixed hormone solutions (*t*_575_ = −1.36, *P* = 0.18). For each sampler type, hormone concentrations differed significantly between samples treated with the mock pregnant female, adult male and adrenal glucocorticoid response solutions, as expected (all *P* < 0.05). However, the accuracy of observed hormone concentrations compared with expected levels was significantly different between sampler types (*F*_3,575_ = 24.09, *P* < 0.001). The resultant hormone measures most significantly affected by sampler type (i.e. *P* < 0.001) were progesterone (low concentration level, *F*_3,60_ = 19.32; high level, *F*_3,28_ = 92.94) and cortisol (low level, *F*_3,28_ = 101.04; high level, *F*_3,60_ = 9.63), as well as estradiol (medium level, *F*_3,60_ = 27.26) and triiodothyronine (medium level, *F*_3,92_ = 62.16; Fig. 2). Across all hormones and concentration levels, results were most accurate from rinsed dishes (range = −3.4 to 2.2 ng/ml), followed by nitex mesh (−4.2 to 3.1 ng/ml), then veil samples (−5.0 to 3.6 ng/ml), and the least accurate results from pipetted dishes (−7.9 to 3.8 ng/ml; *F*_3,575_ = 27.59, *P* < 0.001). The magnitude of change of sample hormone results attributable to the use of each sampler type was greater than the error expected owing to assay performance across runs (i.e. inter-assay coefficient of variation <7%). In addition, the direction of change (i.e. elevated or reduced recovery) in resulting measures for each sampler type also varied depending on the hormone type and concentration level (see Fig. 2). For dish samplers, hormone recovery on average was significantly improved when the EtOH rinse was applied (100 ± 3%) compared with directly pipetting the sample off the surface (88 ± 6%; *t*_44_ = −2.05, *P* = 0.04). In particular, progesterone recovery significantly increased, on average by 81%, when applying an EtOH rinse to the dish surface (*t*_44_ = −17.51, *P* < 0.001; all other hormones, *P* > 0.05; see Fig. 2). Recovery of sampled hormone from the nitex mesh samplers was on average 101 ± 3% and from veil samplers 97 ± 5%.

**Figure 3:**
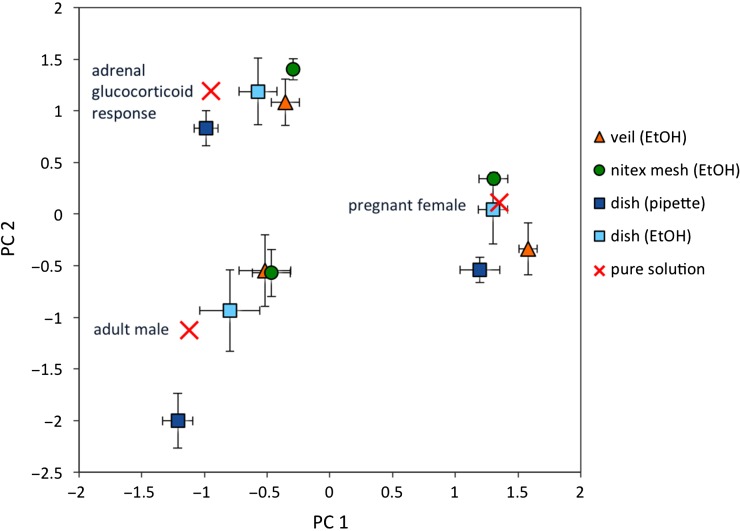
Component matrix of the principal component analysis, showing the factor scores for each treatment solution (adult male, pregnant female and adrenal glucocorticoid profile) after processing on each sampler type [veil (EtOH), nitex mesh (EtOH), dish (pipette) or dish (EtOH)]. Expected levels in pure mixed hormone solutions before processing are marked with a cross. Values are means ± SD.

Principal components analysis incorporating concentrations for all six hormones revealed two components, PC1 and PC2, explaining 43 and 31% of the variance, respectively. Both components showed a number of strong loadings, with five of six variables loading substantially on only one component. Cortisol and progesterone loaded (pattern coefficients >0.91; structure coefficients >0.92) strongly on PC1. Aldosterone, estradiol and testosterone (pattern coefficients >0.67; structure coefficients >0.71) loaded strongly on PC2. Each treatment group for each sampler type was plotted as a function of its loadings for PC1 and PC2 and compared with the expected levels in pure mixed hormone solutions. Multivariate principal components analysis demonstrated clear differences in the hormone profile of each treatment solution before sample processing (Fig. 3). After sample processing on different sampling materials (i.e. including effects of respective containers and extraction protocols), results for each sampler type maintained the distinct variation between treatment solutions. However, rinsed dishes produced more similar results to the levels in pure solution for all treatment groups compared with other sampler types (Fig. 3). Nonetheless, all the tested sampling materials and extraction methods demonstrated good precision in resulting hormone concentrations, as determined by low standard deviations between replicates (Fig. 3).

## Discussion

Measurement of hormones in blow is a promising non-invasive approach to gather physiological data from free-swimming whales. However, reliable sample collection and hormone extraction techniques are pivotal to accurate analysis of blow hormones. Furthermore, these critical steps are also the most labour-intensive component of sample analysis and, possibly, the most error-prone part of the process (Lynch *et al*., 2003). Consequently, advances in blow hormone analysis are contingent on validation and improvements of technical methodologies; this is especially true given the low hormone concentrations in blow samples. Our study demonstrated that three sampling materials have the potential to influence the results of immunoassays for multiple hormone analyses at the levels conjectured for blow. Nonetheless, relative hormone patterns (i.e. low/medium/high levels for all six hormones) and mock physiological profiles (i.e. adult male, pregnant female and adrenal glucocorticoid response) were able to be identified correctly using any of the tested sampler types, suggesting that each sampler type may still be viable for collection of whale blow. Polystyrene dishes proved to be the most effective surface for sample accuracy and precision, yielding measures close to absolute values. However, samples collected on polystyrene dishes had to be extracted with an EtOH rinse, because direct pipetting reduced hormone recovery.

Samples collected in the field can be vulnerable to artificial fluctuations in hormone concentration resulting from less than ideal sample storage and transportation methods (Woods, 1975; Whitten *et al*., 1998; Palme, 2005). Ideally, it is recommended to preserve samples immediately at sub-zero temperatures in order to minimize degradation of hormones (Woods, 1975; Whitten *et al*., 1998). Achieving freezing conditions can be logistically problematic in remote situations (Khan *et al*., 2002; Edwards *et al*., 2014); however, temporarily chilling samples in an insulated cooler is practical for small research vessels at sea. Our results showed that concentrations of six hormones (representing one thyroid hormone and all five classes of steroid hormone) did not change in samples stored in a cooler on ice for up to 6 h, suggesting that cold storage (~4°C) preserved sample integrity for a practical period of time. Previous studies on blow hormones have added a preservative (e.g. manganese chloride) to samples in order to prevent hormone degradation (Hogg *et al*., 2005, 2009). Our results, in agreement with Trout (2008), suggest that the addition of such an inhibitor to stabilize sample hormones may not be necessary. It should be noted that our experiment did not involve the complex biological matrix expected in exhaled breath condensate; in particular, naturally occurring bacteria (Acevedo-Whitehouse *et al*., 2010). The issue of bacterial metabolism of hormone samples is especially marked in excreted faeces, where gastrointestinal bacteria are abundant (Whitten *et al*., 1998). However, faecal hormone samples kept on ice immediately after defecation are considered to be relatively stable during the period of field collection (reviewed by Khan *et al*., 2002). In comparison, plasma steroids are more stable, with no change in concentrations for up to 72 h at room temperature and for at least a week at 4°C (Grant and Beastall, 1983; Bolelli *et al*., 1995). We theorize that hormones in blow are likely to have a stability closely resembling hormones in blood, and conclude that although immediate proper preservation (i.e. freezing samples) at the site of collection is preferable, the basic cold storage equipment tested here (i.e. thick-walled insulated cooler with ice packs) was adequate for blow sample preservation while conducting daylong fieldwork at sea. We emphasize that additional storage regimens, durations (i.e. longer-term storage >6 h) and preservation temperatures could be tested in follow-up studies, but the stability noted here is encouraging for proceeding with an insulated cooler for short-term storage, which is known to be feasible for most boat-based researchers.

Extraction of hormones from the sample matrix is usually the first preparation step in quantifying hormone concentrations in biological samples. Many different extraction techniques (and solvents) have been developed to recover and extract hormone from various sample types (e.g. Rolland *et al*., 2005; Kellar *et al*., 2006; Hunt *et al*., 2014b), so proper selection and testing of the extraction procedure is essential. For the present study, extraction techniques were specific to each sampler type and were intended to maximize hormone recovery, with consideration for logistical efficiency (i.e. processing time and required resources) and sampler design characteristics (i.e. fabric cf. solid surface). Overall, the extraction methods tested in this study yielded valid concentrations of steroid and thyroid hormones in replicate samples, demonstrating consistent and repeatable results for all hormones. However, most notably, hormone recovery was reduced when a pipette was used to collect the sample directly off the dish surface. Our results demonstrate that rinsing the sampling surface with EtOH significantly improved sample hormone recovery and absolute value estimates, especially for progesterone. Progesterone is a less polar compound than the other hormones examined in this study (Westphal, 1986), and molecules may be particularly prone to adhering onto the surface of sampling material (i.e. rather than remaining suspended in the aqueous blow droplets). Hunt *et al*. (2014a) found a similar pattern for hormones in North Atlantic right whale blow that had been collected with nylon veils; in that study, the cortisol content of split samples was always greater when the veil material was rinsed with EtOH than when sample droplets were recovered by pipetting and/or centrifugation of the fabric. As this phenomenon has been now recorded with two different sampling materials, it seems necessary that blow sample processing methods should routinely use a solvent rinse on the sampling device to recover hormones, i.e. an EtOH or other solvent rinse, rather than relying on physical extraction of droplets from the sampler. Although rinsing is more time consuming (because of the dry-down phase) and costly (the chemical EtOH is required), it appears necessary for maximal isolation and/or concentration of the hormones of interest. This may prove crucial, considering that the collected blow specimen will probably be low volume, low concentration and diffusely distributed across the sampling material.

Sampler materials used to absorb blow during sample collection and processing must be evaluated because exogenous substances can interfere with hormone analytical methods, sometimes leading to erroneous hormone concentrations (Shirtcliff *et al*., 2001; Tate and Ward, 2004; Bowen and Remaley, 2014). Of the sampler and extraction processes tested here, the veil and nitex mesh protocols (i.e. the sampler itself and the extraction process used to recover hormone) were more susceptible to apparent (spurious) hormone results in some assays, particularly progesterone, estradiol and cortisol. The container bags and jars, used in conjunction with these sampler types, produced no detectable interference effects when tested in isolation (see ‘*Materials and methods*’). Therefore, it is plausible that the measured immunoassay interference may be mostly attributed to nylon-related substances in the veil and nitex mesh materials, which may have leached into the sample during extraction, affecting antibody binding. Fortunately, immunoassay interference for veil and nitex mesh was consistent and generally low (<0.3 ng/ml in most cases); and, most crucially, these inherent levels did not impede the overall accuracy of hormone results in the sample. Furthermore, expected sample ratios (i.e. low/medium/high concentrations) were still correctly distinguished for all hormones. Thus, the levels of assay interference noted here, for all sampler types tested, can probably be regarded as tolerable ‘background noise’ that did not distort conclusions. Nonetheless, these results should caution investigators that materials (especially nylon) could potentially interfere with immunoassay results, depending on the hormone of interest. This study achieved the first step of demonstrating whether or not such interference occurs at all, but future studies could seek to partition the source of assay interference when further optimizing collection alternatives. We strongly recommend that researchers using novel sampler types should specifically test for assay interference from all exogenous materials used in the collection and extraction process, as well as routinely including ‘blank’ samples in their studies to serve as a negative control (i.e. sampling materials with no blow sample added that have been taken through the entire laboratory extraction process). Ultimately, it is crucial that researchers be aware of the level of hormone assay interference produced by materials used in sample collection and processing, and must carefully evaluate the suitability of new collection materials to ensure that signals of biological interest are still detectable and not misinterpreted.

The discipline of non-invasive field endocrinology is still evolving for marine mammals, and the present study has presented important technical validations for sampling materials, the most practical storage to stabilize samples during daylong fieldwork, and efficient extraction techniques for hormone analysis of blow samples from any whale species. Using the techniques outlined in this study, relative patterns and near absolute values were measured, at levels conjectured for blow samples, for a mix of six hormones of interest in conservation physiology. With this technical information, researchers can make informed decisions about appropriate sampling materials, effective field storage and reliable sample preparation for integrity of blow hormone results. The present study, in combination with work by Hunt *et al*. (2014a), which places emphasis on validating the immunoassay antibodies for a given hormone and species, provides critical groundwork for dependable sample collection and analysis techniques that may ensure reliable hormone data obtained from whale blow. The next essential step for developing blow hormone analysis must be to determine an exact adjustment to correct for the unknown total volume and water content of the blow specimen collected, thereby permitting hormone concentrations in whale blow to be quantified precisely.
